# Drug injecting and HIV risk among injecting drug users in Hai Phong, Vietnam: a qualitative analysis

**DOI:** 10.1186/s12889-015-1404-3

**Published:** 2015-01-29

**Authors:** Tanvir Ahmed, Thanh Nguyen Long, Phan Thi Huong, Donald Edwin Stewart

**Affiliations:** School of Medicine, Griffith University, Griffith Graduate Centre, South Bank Campus, 226 Grey Street, South Brisbane, QLD 4101 Australia; Vietnam Authority of HIV/AIDS Control, Lane 135/3 Nui Truc Street, Ba Đinh District, Hanoi, Vietnam

**Keywords:** HIV, Injecting drug user, Vietnam, Sharing, Qualitative, Harm reduction, Prevention

## Abstract

**Background:**

Hai Phong, located in northern Vietnam, has become a high HIV prevalence province among Injecting Drug Users (IDUs) since the infection shifted from the southern to the northern region of the country. Previous research indicates high levels of drug and sex related risk behaviour especially among younger IDUs. Our recent qualitative research provides a deeper understanding of HIV risk behaviour and highlights views and experiences of IDUs relating to drug injecting and sharing practices.

**Methods:**

Fifteen IDUs participated in semi-structured interviews conducted in September-October, 2012. Eligible participants were selected from those recruited in a larger scale behavioural research project and identified through screening questions. Interviews were conducted by two local interviewers in Vietnamese and were audiotaped. Ethical procedures, including informed consent and participants’ understanding of their right to skip and withdraw, were applied. Transcripts were translated and double checked. The data were categorised and coded according to themes. Thematic analysis was conducted and a qualitative data analysis thematic framework was used.

**Results:**

Qualitative analysis highlighted situational circumstances associated with HIV risks among IDUs in Hai Phong and revealed three primary themes: (i) places for injecting, (ii) injecting drugs in small groups, and (iii) sharing practices. Our results showed that shared use of jointly purchased drugs and group injecting were widespread among IDUs without adequate recognition of these as HIV risk behaviours. Frequent police raids generated a constant fear of arrest. As a consequence, the majority preferred either rail lines or isolated public places for injection, while some injected in their own or a friend’s home. Price, a heroin crisis, and strong group norms encouraged collective preparation and group injecting. Risk practices were enhanced by a number of factors: the difficulty in getting new syringes, quick withdrawal management, punitive attitudes, fear of arrest/imprisonment, lack of resources, incorrect self-assessment, and risk denial. Some of the IDU participants emphasised self-care attitudes which should be encouraged to minimise HIV transmission risk.

**Conclusion:**

The IDUs’ experiences in Hai Phong identified through our data broaden our qualitative understanding about the HIV transmission risk among IDUs and emphasize the need to strengthen harm reduction services in Vietnam.

## Background

Globally, injecting drug use accounts for a high proportion of new HIV infections and continues to have a significant impact on national HIV epidemics amongst Injecting Drug Users (IDUs) [1]. Some countries in East and Southeast Asia face a critical form of the drug use driven HIV epidemic [2]. Although the HIV epidemic in Vietnam is still in a ‘concentrated’ stage, there is substantial risk with an overall HIV prevalence of 20% reaching up to 50% in some places among IDUs [3].

HIV testing in Vietnam started in 1988 and the first HIV infection was detected in 1990 [4]. Since then, the epidemic has progressed very rapidly and this explosive boom was recognized in 1993, especially in southern Vietnam [4]. In that year alone, 945 cases were reported, of which 87% of sero-positive persons were IDUs [5]. By the mid-1990s, the epidemic was well-established among IDUs, prevalence had reached 70-80% in different parts of the country and other risk groups such as Female Sex Workers (FSWs) were being affected. By the end of 2000, the epidemic shifted from the southern to the northern region, infecting younger IDUs [6]. Consequently, the northern port city of Hai Phong, one of the three largest cities in Vietnam with the status of a province, situated in the development triangle marked out by Ha Noi, Hai Phong, and Quang Ninh, has become one of the highest HIV burden provinces [7].

Hai Phong is situated 102 km to the east of Hanoi and 20 km from the sea. The province has enormous economic potential because of its geographic location, economic importance, and its effective national, regional and international transport network including inland water communication. Since the introduction of the open market economic policy (*Doi Moi*), Hai Phong has attracted immense direct foreign investments contributing to the overall development in Vietnam. Over the years, Hai Phong has grown significantly as an economic, cultural, and tourist centre and has attracted many visitors. These significant development changes helped new drug users to emerge and encouraged commercial sex work to flourish [5]. Furthermore, Hai Phong is close to the well-established heroin shipment route which connects selected northern provinces with the ‘Golden Triangle’ (an opium producing zone), creating easy access to an ample supply of heroin at a cheap price [8]. These circumstances provide the worrying potential to intensify the HIV epidemic situation associated with drug use and sex work [9]. Hai Phong has experienced a very high level of HIV prevalence among IDUs. However, there is little in-depth understanding of behavioural risks associated with drug injecting and sharing practices among IDUs in Hai Phong. This qualitative study highlights drug use and sharing practices to understand the potential factors that contribute to the high level of HIV prevalence better.

The first HIV infection in Hai Phong was reported in 1994. According to sentinel surveillance data, the prevalence among IDUs climbed rapidly from 1% in 1997 to 32.8% in 1998 [6]. Reaching a record peak level, the prevalence has currently levelled-off at around 60%, creating one of the highest HIV prevalence provinces in the northern region [7]. The latest Integrated Biological and Behavioural Surveillance (IBBS) survey data reported 48% HIV prevalence among IDUs in Hai Phong [3]. Over the years, other research has also documented high level prevalence, with frequent drug and sex related risk behaviour especially among young injectors [10]. Moreover, unsafe drug and sex related behaviour of HIV infected persons (PLHIV) has heightened the risk of a heterosexual epidemic in the future [11,12].

The HIV epidemic in Hai Phong gained momentum rapidly as a result of the early diffusion among the high risk groups of IDUs and FSWs. In response to both the drug and HIV problems, a number of pilot projects were initiated in various provinces including Hai Phong [10]. A lack of adequate policy support interfered with these ongoing programs. However, following simultaneous change in the national policy and the legal environment in terms of the implementation of a harm reduction program, the provincial response to HIV in Hai Phong has been remarkable [13]. Development of a significant response towards the rising HIV prevalence among IDUs became a shared concern of local, national and international authorities and HIV prevention services have gradually expanded in Hai Phong, including antiretroviral (ARV) treatment opportunities. Also, in 2008 a pilot Methadone Maintenance Treatment (MMT) project was introduced in Hai Phong [14]. At present, the HIV prevention program in Hai Phong has reached a mature stage with high coverage.

Despite this gradual yet substantial HIV response in Hai Phong, the city continues to experience a very high rate of prevalence among IDUs [15]. High risk behaviours of young drug users, frequent mixing with FSWs and continued risky practices by PLHIV have been documented as contributory factors in sustaining the high HIV prevalence among IDUs [7,12,16]. Previous research in Hai Phong has been primarily quantitative and focused on either younger IDUs or PLHIV in order to document ARV adherence or methadone treatment [12,16,17]. Therefore, this research aims to fill the gap in qualitative information and to investigate drug injecting and sharing practices to help generate insights for HIV prevention in IDUs. These qualitative data focus on the experiences and views of IDUs relating to drug injecting and sharing practices and thus provide a deeper understanding of previously unexplored aspects of transmission risk as well as the high HIV prevalence.

## Methods

### Research design

We used an exploratory qualitative research design to gather insights and detailed explanation on drug injecting behaviours associated with HIV risk among IDUs in Hai Phong.

### Study population

A total of fifteen IDUs both male and female between the ages of 25 and 49 years from different districts in Hai Phong participated in this research and thus comprise the study population. Inclusion criteria highlighting features such as age, sex, risk characteristics/profile, were followed in order to obtain a range of information. Eligible participants (except two female respondents) were a sub-set of individuals selected from those recruited for a larger scale national level behavioural research initiative, during which a field supervisor from the provincial harm reduction program had asked screening questions and ensured recruitment status.

### Sampling

The objective of this research was to gather qualitative perspectives and contextualise in-depth understanding about heroin injecting, sharing, and associated HIV transmission risk among IDUs in Hai Phong. Therefore, the focus was the quality and content of interview rather maximise the number of interviews [18]. Accordingly, we employed an opportunistic sampling approach to recruit study participants. A peer educator was involved with the recruitment of the study participants and had accessed a number of social networks of IDUs before the interview. Members of these multiple networks were invited to participate in the exploratory qualification research. In addition, we encouraged participation through a snowballing approach after the interview of each participant.

### Research instruments

A semi-structured interview checklist was prepared in line with study objectives. A facilitator’s guide was also developed to identify necessary probes and different stages of probing to complement the interview checklist. The checklist was shared with persons who work at field level to ensure that it would capture information on relevant aspects. The instruments with guidelines were translated into the Vietnamese language, discussed thoroughly with field workers and tested to validate the language, content and order. The interview checklist included the following topics: drug use behaviours, sharing practices, condom use status, access to HIV prevention services and finally participant’s recommendations.

### Data collection

The research was conducted during September and October, 2012. A semi-structured, face-to-face qualitative interview technique was used to collect detailed information on risk behaviours associated with heroin injecting. The facilitator’s guide with explanations under each theme provided guidance to the data collectors. Two local interviewers conducted the interviews in Vietnamese. The interviewers were employed in a local research organization and had adequate knowledge about the IDU population and local drug use scenario. They were also experienced in collecting qualitative data. Furthermore, the first author briefed them on the research objectives, discussed the research instruments in detail and clarified different probes, times, and styles of probing to generate discussion with participants. The interviews were conducted in a friendly environment (calm, private, free from any distraction) allowing participants enough time to express their thoughts at some length which the interviewers recorded comprehensively [19]. The interviews generated a lot of discussions surrounding the research topic which sometimes went beyond interview content but any emerging issue raised in one interview was covered in subsequent interviews with other participants for more in-depth understanding. This helped to gather information on emerging topics to supplement the analysis stage. Lastly, attention was paid to identify the level and point of information saturation, to determine a possible end for interviews [18]. The interviews lasted for about forty-five minutes.

### Data management and analysis

The interviews were audio taped with permission. All study materials (such as audio files, interview scripts, and consent form) were assigned unique identification numbers and then edited to remove all personal identifiers. The interview scripts were transcribed from audio format to paper file. The transcription process lasted for around three weeks and during this process, the researcher (first author) and two interviewers were actively involved and monitored constantly. Later, the interview transcripts were translated into English with double checking. Continuous checking and re-checking was performed during the translation process to detect any inconsistencies and misrepresentation. This was repeated to confirm the meaning and context of original narratives and to finalise the translated interview scripts for data analysis.

Qualitative data analysis was performed manually. All the transcripts were read thoroughly to understand the main context of each interview, followed by detailed examination based on the study objectives. Then data were categorized and coded into themes reflecting the research objectives, with the topic guide and narratives of the participants used for framing codes and themes. Thematic analysis was used to identify, analyse and report different themes into textual data [20] and a qualitative data analysis thematic framework was adopted [21]. Key themes were compared across transcripts to identify consistency throughout the exploratory quotes. The views and experiences of the participants regarding the recurring themes/sub-themes are presented with exploratory quotes in the text showing the number of the interview case within parenthesis. A schematic diagram is presented highlighting major themes and recurring sub-themes to show relationships, direct or indirect, associated with drug injecting and sharing practices.

### Supervision and quality control

The first author was actively involved in the research and directly supervised the data collection process. A team consisting of a peer educator from a provincial harm reduction program and a member from a local research organization accompanied the researcher to different hotspots to gather knowledge on drug settings. Interview sessions were monitored and included discussion with interviewers after each interview and checking for completeness and consistencies to ensure data quality. Immediate discussion with interviewers after each interview and writing of interpretive notes enhanced understanding and facilitated analysis at a later stage. Data analysis was performed manually after which this manuscript was drafted.

### Ethical procedures

The research was conducted following ethical clearance obtained from the Office of Research at Griffith University. Authorisation also was received from the Vietnam Authority for HIV/AIDS Control (VAAC). The consent form, interview checklist and facilitator’s guide were translated into Vietnamese. Participation was voluntary and anonymous. Before the interview informed consent was explained to the participants as well as their right to withdraw, skip or refuse to answer at any time during the interview. They gladly expressed their interest in participation and provided written consent. The choice of a private location for the interview was also convenient for respondents. Information gathered was treated as confidential and only accessed by the principal investigator who strictly monitored the transcription and translation processes to ensure data security. Lastly, the participants were reimbursed VND 100,000 (about AUD 5) for their time and any inconvenience experienced.

## Results

### Profile of participants

The socio-demographic profile of the participants is provided in Table 1. Of the 15 participants 13 were male. The two female injectors were also FSWs. Among our IDU participants, nine were young adults (aged 30–39 years), with four younger (less than 30 years) and two older (40 years or more). All the participants belonged to the ‘*Kinh*’ ethnic group and all except one were permanent residents in Hai Phong province. Similarly, all except one were long term residents in Hai Phong. Thirteen participants had completed primary or secondary school and two had completed college or university level education. Seven participants were currently married, one was living alone and the rest were either living with their wives and children or parents and other family members. Three participants were unemployed and the remaining 12 were employed in some form of non-regular unstable casual work, such as motor bike driver, mechanic, or small informal business. Unemployed participants mostly relied on family support. Employed participants mostly earned less than five million VND per month (8) and only four earned an average five million VND or more. In terms of overall family income the majority (12) earned less than ten million VND a month (just less than AUD 600).

**Table 1:** **Socio-demographic characteristics of respondents**

**Characteristics**	**Categories**	**Number (N = 15)**
**Gender**	Male	13
	Female	2
**Age** (range: 25–49 years)	Less than 30 years	4
	30–39 years	9
	40 or plus	2
**Ethnicity**	Kinh	15
**Place of living**	Hai Phong	14
	Other province	1
**Duration of living in Hai Phong**	Permanent	14
	Temporary	1
**Education**	Primary/secondary	13
	College	2
**Marital status**	Currently married	7
	Unmarried	8
**Living status**	Co-habiting	14
	Alone	1
**Employment status**	Casual/non-regular work	12
	No work	3
**Income level** (range 2 m-7 m)	Less than 5 m VND	8
	5 m VND or more	4
**HIV status**	Positive	6
	Negative	9

Among the 15 participants, six, all male, were infected with HIV. Most of the HIV infected participants became aware of their status between 2006 and 2010. Five of the participants were registered with clinics and had already started ARV treatment and one had not registered with any clinic. One of them had also started methadone therapy in 2012. All the HIV infected participants identified frequent sharing of needle/syringe (N/S) and other injecting equipment (such as water, common containers, cotton) as the mode of acquiring the virus.

### Drug use behaviours

Context of first time drug use behaviour and place for injecting has been reported as key themes under drug use behaviour. The following section extends the views and experiences shared by participants surrounding these two themes.

#### Context of first time drug use

Our study group was comprised of participants of different ages and we were interested to understand the context of engaging in drug use behaviour for the first time among younger and young adult IDUs. Therefore, the participants were asked to describe in detailed their first experience of drug use. A number of factors including personal, social, and external environmental conditions were identified as reasons for their first experience of taking drugs. The majority of the younger IDUs (less than 30 years) started using drugs because of peer pressure, as one person in a group starts taking drugs, other friends followed on in order to sustain the friendship. The comments made by the majority of the young adult IDUs (30–39 years) can be grouped as ‘personal circumstances’ where they mentioned ‘curiosity’ and ‘lack of awareness’ regarding drug use behaviours and its adverse effect. Some of the younger participants highlighted ‘existing social circumstances’ such as drugs introduced by close friends in disguise (without informing) and ‘external environment conditions’ such as family history of selling heroin had an influence on their initiation to drug use. In addition, first time experimentation often arose out of a desire for fun and pleasure for a few younger IDUs. One older participant (above 40 years) mentioned that being ‘ignorant’ about drugs led to his first time drug use. All interview participants described either a poor or middle-level socio-economic profile. The social context [22] had played a significant role among younger IDUs in their initiation to drug use.

Almost all the participants began using drugs by inhaling opium, then smoking heroin and later gradually moved to injecting, especially heroin injecting. A transition in drug injecting took place during the mid-1990s when injecting heroin replaced injecting ‘black water’, a concoction made from a residue of opium prepared for smoking. Some of the young adult participants (30–39 years) discussed this shift in drug use and mentioned that because of ‘easy accessibility of heroin’ at a ‘reasonable cost’ as a result of frequent supply from the ‘Golden Triangle’ zone many of the IDUs resorted to heroin injecting. The participants mentioned that it took between two and six years for most of the IDUs to switch from non-injectable to injectable drugs. Heroin is the most cited and preferred injectable drug in both Hai Phong and nationally due to its easy availability at a reasonable price. Some mentioned that to celebrate special event, religious festival or friend’s birthday, for example, they occasionally tried other types of drugs (Ecstasy, or ATS). Only a few of our participants mentioned that they had used other drugs concurrently in the month prior to the interview.

#### Places for injecting

We were interested to expand our knowledge about the risk environment generally associated with Vietnamese IDUs so they were encouraged to discuss the places they prefer to inject and situational circumstances they face. Our participants described the places where they took drugs in detail. A number of hotspots near rail lines were identified as the most popular places for buying and injecting heroin in Hai Phong, although currently there is no fixed place, since it moves along the rail lines from time to time to avoid police attention. Later, they highlighted risks associated with injecting in these places.

All the participants mentioned that the police are very strict and perform frequent raids (crackdowns) along the rail lines and therefore, the IDUs did not gather in big numbers in a fixed place. Many of them just went at a specific time of the day so that they did not draw public attention. Police attention along the rail line has been on-going for the last two years and currently there is a supply crisis. The comments made by two interviewed participants reconfirmed the fear of ‘police arrest’ in places adjacent to rail lines and voiced their intention to ‘avoid police harassments’ by injecting in another hotspot such as under the ‘*Niem’* Bridge which is a quiet place attracting few people. They increasingly used different places other than rail lines, such as their own home or a friend’s house, a deserted street, alleys, or parks for injecting. Many of the participants contacted the seller by mobile phone and got the delivery at a point near their home. According to the participants, injecting in their own or a friend’s home helped promote safe practice, unlike streets or public places where they were required to ‘inject in a hurry’ and to manage ‘withdrawal quickly’. The participants emphasized their reluctance to carry extra syringes as this would provide incriminating proof to the police and result in detention. These findings confirm the on-going ‘punitive approach’ maintain by police that interacts negatively with the risk environment and facilitates transmission risk [23].

Often the sellers did not allow IDUs to inject near the rail lines because this could then draw the attention of police making it difficult for the sellers to operate their business. For this reason sellers only sold drugs at certain times each day and required IDUs to visit the rail lines at that particular time of the day to buy them. However, despite police raids and the risk of getting caught, the areas in the vicinity of the rail lines were still the preferred places for injecting for most IDUs. They avoided taking drugs near the rail lines because of the ‘fear of the police’ however, when they bought drugs near rail lines often they took a chance and many IDUs did in fact inject drugs there.

### Group injecting

Our participants mentioned the development of small groups or cliques while discussing IDU networks and this group injecting behaviour became another major theme in our analysis. It frequently occurs with drug using peers who share common behavioural traits, mutual economic ties and social bonds and often develops into drug related partnerships [24,25]. The following section highlights group injecting behaviours in the context of Vietnamese IDUs.

Group injecting appeared to be very common for most IDUs and all of the interviewed IDUs frequently participated in group injecting. Generally, small groups with two or three persons who were very close engaged in group injecting. One respondent described his last injection episode in groups:

*We (respondent with two friends) gathered our money and bought a (heroin) pack costing 300,000 VND. One among us prepared the stuff (liquid solution) using purified water. He used a new syringe to put it all and mix together. After complete mixing we used new syringes to divide the liquid drug into three (back loading) (c1)*.

Group injecting behaviour is closely linked with shared use of drugs which involves sharing the N/S or other injection paraphernalia and which inadvertently becomes an HIV transmission risk [26,27]. The risk associated with shared use of drugs is expanded below in the ‘sharing practices’ section.

The price of drugs and the money available to IDUs together generate the greatest economic motivation for group injecting behaviour. The price of a heroin pack fluctuates and when the price is high IDUs did not have enough money to buy them individually. As an alternative option they bought them jointly and then divided the drug. The IDUs often considered the shared use of drugs to be an opportunity for them because this helped them to take drugs needed them, in spite of not having sufficient money. Often police raids and severe law enforcement activities, such as massive search operations, created a crisis in heroin supply leading IDUs to buy drugs jointly and inject in groups. According to one participant:

*I do not have a few thousand always with me to buy this (heroin). So I want to meet them (my friends) more often and buy things (heroin) together. My friends are also like me (they also want to meet). (If not find me) they buy with someone else (c3).*

Another aspect of group injecting, as highlighted by many of our participants related to ‘norms and friendships’. There are strong bonds among drug using friends and they like to take drugs, mingle with one another, and enjoy different events together. Also, a group norm develops, intensifying their intimacy and friendship as they experience the same drug taking events together.

### Sharing practices

Sharing practices are based on deep social and cultural norms and values which continuously influence the risk engagement of IDUs [28]. They experience sharing as a ritual that acts to make their friendships closer and strengthen the bonds between them. Unfortunately, sharing practices (including sharing injection paraphernalia) play an important role in HIV transmission among IDUs [29] when needles/syringes are shared directly, for example, by giving their own personal N/S to a group member after using, or receiving the same after another group member had used, thus contributing to ‘higher risk’. There are also risks from indirect sharing, for example, by sharing common water containers, drug solutions, cotton or even not using a new needle/syringe during the preparation stage of the liquid drug solution, thus contributing to ‘lower risk’ [27]. The process of drug sharing often involves indirect sharing because of the embedded mechanisms of sharing techniques commonly known as either ‘frontloading’ or ‘backloading’ [28].

According to research evidence, sharing behaviour has been widespread among IDUs since the inception of the epidemic in Vietnam [10,17,23,30,31]. However, because of improved knowledge, IDUs are now more aware of HIV transmission risks and are more prepared to avoid sharing practices [32]. Our interviews included in-depth discussions of both direct and indirect sharing practices and the following section highlights different types of sharing practices, the reasons why they shared and participants’ knowledge about sharing risks.

#### Sharing needles/syringes

Our qualitative data reaffirmed evidence [32] that indicated that the prevalence of direct sharing is very low because of improved knowledge. According to the comments of some participants they are now willing to buy new N/S when they buy the heroin pack. One participant said:

*There are some people who sell new needle/syringes in the gathering places. Those who sell drugs and sell new needle/syringes in the rail lines are different. If we do not find them (peer educators) we can buy new needle/syringes at any drug store along the rail lines (C14).*

Another participant confirmed this with a further comment about not engaging in direct sharing:

*Sometimes I buy this (heroin) with my friends, mix it in the new syringe and then divide. I do not share needle/syringes. I do not give my used needle/syringes to others (c12)*.

These consistent statements support the contention that there has been a change to non-engagement in direct sharing behaviour [17,23,30]. However, this contention must be qualified based on further discussion of a number of circumstances and contexts relating to their sharing practices.

#### Sharing drugs and injection paraphernalia

Shared drug use during group injecting is common because of the situation that IDUs face, as identified above. One participant, for example, described the process of shared drug use during group injecting, saying:

*At first we buy the drug (one pack heroin) jointly each contributing equally (for buying one pack of heroin worth 100,000 VND we contribute equally 50,000 VND). Since we contribute equally we also share the drug equally. It is very difficult to share the pack equally. Therefore, we need to dissolve the drug by mixing purified water in a syringe and prepare the liquid drug solution reaching to the 10 ml level. Then we can share equally 5 ml by transferring (liquid drug solution) in another syringe (c2)*.

A range of risk behaviours take place through indirect sharing of injection paraphernalia when IDUs prepare, measure and distribute such a jointly purchased drug and thus highlight the possibility of HIV transmission [24]. The environmental circumstances of the places where they inject do not facilitate a safe drug sharing process and involve risky injection among the members [23]. The view of one of the HIV infected participants on sharing behaviour as the reason for contracting HIV was that:

*I shared drugs most of the time in groups. We did not use new needle/syringe for preparing drug solution and dividing amongst us every time. This is really very difficult to make sure the new needle/syringe every time and remain careful in using container during preparing and dividing the combined drugs. This has been the reason for my infection (c10).*

Another participant focused on a set order or procedure in injecting shared drugs during the group injecting process. The ownership of new N/S and the capacity to purchase the drug determined the sequence of injecting. He said:

*Sharing mixture using old syringe (used) is not safe. If there is only one syringe then one person needs to share after the use of the other. In this case the person who is the owner of the syringe would inject first. I bought this stuff and I injected first and then I gave to my friend the needle/syringe and some mixture also (c2).*

Generally the hygiene practices associated in group injecting for most of the IDUs were poor and thus inadvertently increased the transmission risk among group members. During the discussions, some participants mentioned re-use of their personal N/S without proper cleaning (not bleached or boiled, just rinsed with water) in many instances to save extra money which require to purchase new syringe during the time of buying drugs. Many did not seem to understand the importance of a safe cleaning process and the risks caused as a result of using a blunt needle. Additionally, carrying personal N/S would risk those IDUs facing police harassments under search operations.

#### Knowledge of transmission risk

Although some IDUs claimed that they have knowledge of HIV transmission risks due to indirect sharing, this seems to be inadequate. Many of the participants used new N/S, but did not perceive other injecting equipment used for drug preparation and dividing to be potentially harmful. They are thus prone to transmission risk through indirect sharing. One said:

*We use new needle or syringe while we inject in groups. We are afraid and always careful that one person’s blood does not get in contact with other person’s. We use our syringes very carefully (c15).*

The same thing was repeated by another female participant. She said:

*I think sharing needles/syringes can cause infection. There are other diseases also. So I am very much afraid of being infected with these diseases because of direct blood contact. I think sharing drugs is not a problem (c5).*

The focus of harm reduction messages should therefore be not only on the broad issue of the infection risk from sharing behaviour, but rather such messages should be more specific, highlighting the different stages of drug sharing and the risks associated with injection paraphernalia.

#### Reasons for sharing

We asked the participants about their reasons for engaging in sharing behaviours and this revealed two predominant explanations which were consistently repeated: ‘difficulty in finding new needles/syringes at the time of need’ and ‘a crisis period either in heroin supply or a personal crisis’. The IDUs also mentioned other reasons which significantly influenced sharing practices including ‘lack of resources’ and ‘quick withdrawal management’. Another participant highlighted the ‘punitive attitude’ of police and others and mentioned:

*… Another reason (second reason) for sharing needle/syringes and other items is pressure from police, guards, and local people. They chase after us every time. So we just want to quickly use this after getting and then leave the place (c10)*.

These reasons were very common and are also found in other countries [33,34]. One of the participants recalled frequent sharing episodes during the ‘time of imprisonment/rehabilitation’ and described the sharing practices in detention centres:

*When I was in the rehabilitation centre I shared a lot there. There was only one needle/syringe and we were many in the centre. We had to share after using/injecting (one after another). During the time in jail we shared without thinking. Outside jail everyone supports using separate needle/syringes. No one supports sharing behaviours (c11)*.

When probed about sharing practices, some IDUs acknowledged ‘lack of knowledge’ as a reason for their continuing engagement in sharing practices. This lack of knowledge about HIV transmission and prevention was indicated in statements that commented on their ‘lack of awareness’, ‘risk denial’, and ‘wrong self-assessment’ regarding the possibility of contracting HIV. All these reasons amplified the transmission risk from sharing practices among IDUs.

### Views and attitudes towards sharing

The participants expressed different personal views regarding sharing practices. They mentioned that the use of a new N/S during injecting shared drugs reduces the possibility of contacting the virus. However, apart from using new N/S during the preparatory stage, shared drugs can be contaminated because of the process used with other injection paraphernalia [26]. According to one participant:

*We do not support sharing needle/syringe and even reusing my own stuff. After use the needle becomes blunt. When one injects with this type of blunt needle to the vein, the tissue in the vein gets damaged and makes it difficult next time and causes injuries and other problems in the body (abscess) (c10)*.

These attitudes of our participants towards reusing needles/syringes highlighted an indirect motivation towards safe practices. Harris and Rhodes [35] have previously reported that venous access and care motivated a number of long term heroin addicts to use new needles to minimise the pain and suffering of difficult injecting episodes, which ultimately helped them avoid hepatitis C infection. The self-caring attitude reflected in this research is new in the context of Vietnamese IDUs and should be utilised to rearticulate harm reduction messages. Such messages could highlight short term benefits like positive vein care, preserving peripheral veins, avoiding riskier injecting sites in the body rather long term harms and thus minimise the HIV transmission potential [36].

The attitudes and perceptions of drug using friends were very important. IDU networks shared common characteristics and had mutual interests. The psychological contexts such as peer norms and lack of self-efficacy often promoted group injecting behaviour and influenced sharing behaviours [37]. Highlighting this issue one participant said:

*I know the attitude of my friends. They are like me. If they do not have money, they have to find a way to find some others and take in groups. If they have money they will also not share. They will take alone (c7)*.

Participants already infected with HIV described a sense of protecting their community and seemed to have adopted different types of management strategies, such as avoiding injecting in groups or always being the final injector. One participant said:

*Since I am now infected, I generally avoid taking with my friends. When (if injecting in groups) I take this (heroin) in the rail lines I break my needle and destroy the syringe. When I have some friends I push (inject) last and do the same thing for protecting others. I do not want others to become infected like me (c11)*.

This was supported by another participant who said:

*After using (injecting heroin) I throw away my stuff (used needle/syringe) so that no one finds it. I do not give mine to others (c4).*

However, some keep them for re-using later, as mentioned by another participant:

*I just use my own. After use then I keep them for the next time I inject (c6)*.

Another HIV infected participant highlighted that many HIV infected IDUs were now very cautious regarding HIV transmission and are willing to save others. He explained:

*In the past people used to distribute their used syringes but now many of the IDUs do not distribute even after asking and begging by other IDUs (who would not able to obtain new N/S) (c7)*.

### Recommendations made by IDU participants

The participants discussed issues which could increase the engagement of IDUs in current harm reduction programs and make a number of recommendations. The primary recommendation emphasized the need to extend the outreach coverage of current harm reduction programs by peer educators. For example, one participant mentioned:

*Increase the number of peer educators and volunteers for the free distribution of needle/syringes and condoms. The new peer educators and volunteers will visit to all the gathering places and could distribute at the time of need (c14).*

They also indicated the importance of making fresh needles/syringes available at night. One participant suggested:

*We can get the new needle/syringes any time in the day time. But this is very difficult to get at night. It is difficult to buy this from drug stores at night also (c6).*

The recommendations which came out of discussions relating to program operation issues included: more awareness building programs and an increase in the number of peer educators so that they could visit multiple injecting sites, or hotspots to distribute service products.

Another important area of recommendation related to strengthening the management strategies maintained by some HIV infected IDUs. Participants thought that these should be widely disseminated so that the virus could not be transmitted to others when group injecting, or engaging in occasional sharing in unavoidable circumstances. According to some of our HIV infected participants many other HIV infected IDUs regularly engage in group injecting. Separate training curricula needs to be developed for HIV infected IDUs to impart such skills and apply in different socio-cultural contexts. They also suggested developing a special program for the HIV infected IDUs and different programs to support the families of HIV infected IDUs.

Another area of recommendation can be derived from the statement showing self-care attitudes by some of the participants. The self-care practices reflected by some IDUs regarding not injecting with blunt needles should be encouraged. This will assist IDUs to get immediate benefits by avoiding risky injection and preserving their serviceable veins which would minimise needle injuries. HIV prevention programs should highlight messages showing the importance of positive vein care so that IDUs in Vietnam have a better chance of having a safe injection.

## Discussion

**Figure 1:**
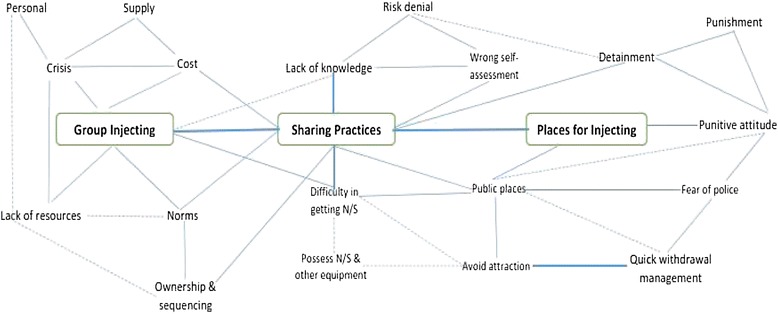
**HIV transmission risks: group injecting, sharing and places for injecting.** Commentary: Three major themes “group injecting” “sharing” and “places for injecting” inter-relate with other recurrent sub-themes showing a direct relationship (by straight lines) and indirect relationship (by dotted lines). Overall, this presents situational and environmental circumstances associated with drug injecting, sharing and HIV risks.

Our qualitative analysis displayed an interrelated picture of injecting jointly purchased drugs in small groups in public places, followed by sharing episodes (direct/indirect). They also highlight the influence of ‘places for injecting’ as an important situational factor facilitating sharing practices among IDUs in Hai Phong. The risk production associated with drug injecting and sharing practices among IDUs as a result of the risk environment was conspicuous in our findings [38] and this underscored the importance of an enhanced harm reduction program to reduce HIV infection by adopting the risk environment approach [39]. A harm reduction program with a social science basis would address the social and environmental conditions identified in this research and significantly benefit HIV response in IDUs. Furthermore, the recommendations emphasized by the participants to maximise their service engagement indicated the need for a comprehensive harm reduction program by improving operation and management. Figure 1 presents our major findings on drug injecting, sharing, and associated HIV transmission risks on the basis of three primary themes: group injecting, sharing practice, and places for injecting. Most of the recurring sub-themes interact with these themes, in most cases directly but sometimes indirectly. This interlinking set of factors influence the risk environment where IDUs inject and thus perpetuate high HIV transmission risks among IDUs in Hai Phong.

Our findings revealed that group injecting behaviour appears to be common among IDUs in Hai Phong and that sharing injecting equipment was routine within social groups [24]. Places visited repeatedly for injecting became one of the major analytic themes, imposing a disadvantageous situational condition which affected and facilitated such sharing practices [34]. Previous research in Vietnam has documented the social and cultural contexts of risk engagement and the social injecting process, where IDUs gather in small groups, jointly purchase drugs by pooling money, and inject in groups by sharing equipment [40,41]. Our qualitative findings confirmed this research, with evidence of similar sharing features associated with drug acquisition in groups, coupled with the strong influence of environmental conditions such as places where IDUs inject and places where the police raid or perform crackdowns frequently.

Sharing practice in groups is consistent with a large body of existing literature [26,31,37,42]. Our findings indicated direct relationships between each of the sub-themes that emerged from social drug using behaviour and sharing, including: impact of a supply crisis, cost of a heroin pack, group norms/friendship, and difficulty in obtaining new needles/syringes. Personal crisis (family situation, withdrawal), lack of resources (inability to buy new needles/syringes), and lack of knowledge of HIV prevention and transmission were found to be indirectly related to the group injecting process. According to the consistent statements of participants, group injecting is a social behaviour for a majority of IDUs and is most likely considered indispensable because of the everyday practical situation they face relating to their drug use [24,42]. A noteworthy finding about the social injecting process was the management strategy adopted by some of the HIV infected IDUs, who either often avoided injecting in groups, or injected last if they did. They seemed to be cautious regarding HIV transmission and acted genuinely to protect their community. This finding was encouraging because a high proportion of HIV infected people had been previously involved in sharing practices, which was an issue of serious concern [11]. Other research provided evidence that HIV infected people including IDUs also adopted protective sexual practices, because of improved knowledge [43].

Our qualitative analysis highlighted a potential indirect transmission risk similar to injecting shared drugs [27] due to the use of common equipment or injecting paraphernalia as part of IDU social drug using behaviour. Improved awareness gathered over many years helped IDUs to adopt self-initiated risk reduction, which resulted in a decreased prevalence of direct sharing [44]. However, evidence shows that while indirect sharing (sharing common injecting equipment) had already been identified as a risk factor for HIV infection among IDUs in Vietnam [31], however, the sharing practices were largely unknown. We have gained important insights regarding sharing practices and revealed a number of contexts (direct or indirect) which interplayed to sustain HIV risk among IDUs. Principal among these were ‘lack of knowledge’ and ‘difficulty in obtaining new needles/syringes’ which have direct relationships with sharing practices and heighten HIV transmission risk. We have found that having a limited knowledge of HIV transmission and prevention was connected with other related outcomes. We analysed the statements highlighting lack of knowledge and found that it caused ‘risk denial’ regarding certain transmission concepts/modes which negatively impacted on IDUs and resulted in an incorrect ‘self-assessment’, which ultimately led to risky sharing practices. Similarly, situational unavailability [45,46] was found to be an additional structural condition which did not facilitate safe drug injecting practices among IDUs, rather it negatively influenced the overall risk environment [38]. We consider it important to highlight two related issues: ‘possession of N/S’ and ‘public place’, which were mentioned repeatedly by IDUs during interviews. IDUs expressed a strong reluctance to carry additional N/S because of frequent police arrests where possession of injecting equipment would be evidence for arrest as a drug user.

Another major theme that emerged in our study related to the places for injecting which IDUs frequently visited. In general, public places such as rail lines, streets, parks and under bridges were the most cited public places. Principal among these was the rail lines and vicinity. These were also the places where police frequently performed massive search operations for narcotics. People found carrying drugs or injecting equipment were humiliated publicly, often beaten or punished in some other harsh way [47]. Hotspots near rail lines kept moving in the face of such crackdowns. Participants highlighted the risks associated with quick injecting while taking drugs in public places, as they try to avoid the attention of police or local people [7]. Other research has indicated ‘place’ as one of the major analytic themes which damage the capacity of IDUs to engage in safe practices through available harm reduction programs [48].

A ‘punitive attitude’ played a critical role in the continuation of sharing practices both in public places and in rehabilitation or treatment centres. Among the IDUs arrested for drug related crimes, a majority after detention were sent to drug treatment centres. Participants discussed drug injecting practices with a higher prevalence of sharing equipment in such treatment centres. The detailed description provided by participants emphasise that risk practices were increased because of injecting equipment unavailability and punitive law enforcement attitudes. The punitive drug policy approach has been criticized internationally because of a series of human rights violations associated with public humiliation, arbitrary detention, inhumane punishment as well as extreme therapeutic treatment processes in these treatment or rehabilitation centres [14,49-51]. Vietnam is currently facing the challenge of successful transition from compulsory treatment centres to a voluntary and community based system [52]. A number of countries have already adopted a favourable drug policy by decriminalising or allowing a threshold level of personal use [53]. A policy change in Vietnam to treat IDUs as people with a health problem has been progressing slowly but is promising [47].

A number of limitations related to these qualitative findings should be taken into account before interpretation [54]. First, our study was designed to capture qualitative perspectives associated with heroin injecting, sharing and transmission risks among IDUs. These qualitative findings are unlikely therefore to be generalizable to the entire population, because of the characteristics of our sample. Our participants came from those recruited from a broader research project with male IDUs. However, in terms of breadth, our project accessed a number of social networks of IDUs in Hai Phong which contained a diverse population (Table 1) and provided a picture of drug injecting and sharing from a range of perspectives.

We do not consider that there was a social desirability bias in our findings because of the minimal involvement of service providers in our research. They only provided assistance to select interview participants. Our semi-structured interview checklists, guidelines and related modifications were discussed thoroughly during field testing and found appropriate to capture the required information. Furthermore, the interviewers were experienced in conducting in-depth qualitative interviews, which facilitated elaborate discussion and thereby minimized the possibility of information bias and chances of misinterpretation in understanding verbal and non-verbal messages. Finally, the information rich content and narratives generated were systematically analysed through a thematic framework. In addition, the first author had active oversight of the data collection process and checking on a day-to-day basis, which enhanced the quality of information and helped contextual analysis of related features.

Despite these limitations, our descriptive evidence as well as the recommendations mentioned by participants provided insights helpful to expand qualitative understanding of the risk environment in a Vietnamese IDU context and underscored the urgent need to strengthen the existing harm reduction services. The participants suggested some operational and management issues to increase service engagement and reduce sharing practices, such as increasing the number of peer field workers and extending field hours, especially at night.

## Conclusion

This qualitative research study identified the experiences of IDUs and the contexts and procedures relating to their drug injecting and sharing practices. It provided evidence of transmission risks which have the potential to exacerbate the current HIV epidemic among IDUs in Hai Phong. The social settings where IDUs frequently injected, such as public places near rail lines, streets, and parks were particularly important in triggering sharing practices. Also, speedy injecting episodes in crisis circumstances (such as withdrawal, crackdowns) elevated the risks as IDUs sought to avoid the attention of police and arrest. This may perpetuate the likelihood of HIV transmission. The impact of repressive policing on sharing practices was emphasised by participants and this had a clearly visible negative impact on the public injecting environment. A change in punitive attitudes and drug policies should allow the urgent shift required to incorporate public health focused activities. The implementation of drug policy recommendations and improved harm reduction services, consistent with other research, would benefit IDUs in minimizing risky practices. Harm reduction programs should be strengthened to increase service engagement with safe injection equipment, and to provide more education about indirect modes of HIV transmission.
